# Short-chain fatty acids are potential goalkeepers of atherosclerosis

**DOI:** 10.3389/fphar.2023.1271001

**Published:** 2023-11-13

**Authors:** Yu Feng, Danyan Xu

**Affiliations:** Department of Cardiovascular Medicine, The Second Xiangya Hospital, Central South University, Changsha, Hunan, China

**Keywords:** SCFAs, gut microbiome, immune cells, endothelium, atherosclerosis

## Abstract

Short-chain fatty acids (SCFAs) are metabolites produced by gut bacteria and play a crucial role in various inflammatory diseases. Increasing evidence suggests that SCFAs can improve the occurrence and progression of atherosclerosis. However, the molecular mechanisms through which SCFAs regulate the development of atherosclerosis have not been fully elucidated. This review provides an overview of the research progress on SCFAs regarding their impact on the risk factors and pathogenesis associated with atherosclerosis, with a specific focus on their interactions with the endothelium and immune cells. These interactions encompass the inflammation and oxidative stress of endothelial cells, the migration of monocytes/macrophages, the lipid metabolism of macrophages, the proliferation and migration of smooth muscle cells, and the proliferation and differentiation of Treg cells. Nevertheless, the current body of research is insufficient to comprehensively understand the full spectrum of SCFAs’ mechanisms of action. Therefore, further in-depth investigations are imperative to establish a solid theoretical foundation for the development of clinical therapeutics in this context.

## 1 Introduction

Coronary artery disease (CAD) is one of the most common causes of human mortality worldwide, of which atherosclerosis (AS) is the main pathological basis.

In recent years, the gut microbiota has been found to play multiple key roles in maintaining host health, including gut homeostasis, immune system response, and protection against pathogens (Hooper et al., 2012). Increasing evidence suggests that the gut microbiota, through metabolites such as bile acids, short-chain fatty acids (SCFAs), lipopolysaccharides (LPS), trimethylamine-N-oxide (TMAO), etc., play a crucial role in cholesterol metabolism, oxidative stress, inflammation, and other fundamental metabolic processes (Cani et al., 2007; Wang et al., 2011; Tang and Hazen, 2014; Jandhyala et al., 2015; Korem et al., 2015; Wahlström et al., 2016; Yissachar et al., 2017). This microbial influence contributes significantly to the occurrence and progression of atherosclerosis. Among these metabolites derived from gut bacteria, SCFAs have drawn extensive attention as potential therapeutic agents for AS (Hu et al., 2022; Lu et al., 2022).

Therefore, this review aims to provide an overview of the latest research findings on how SCFAs can ameliorate atherosclerosis risk factors and elucidate the molecular mechanisms that regulate endothelial and immune cell functions. Furthermore, we seek to unveil the potential of SCFAs in the context of preventing and treating atherosclerosis.

## 2 Short-chain fatty acids

SCFAs are carboxylic acids with aliphatic tails of 1–6 carbons of which acetate (C2), propionate (C3), and butyrate (C4) are the most abundant produced by anaerobic fermentation of dietary fibers in the intestine (60:20:20 mM/kg, respectively, in the human colon) (Makki et al., 2018; Macfarlane and Macfarlane, 1997; Cummings, 1981; den Besten et al., 2013). The type and quantity of SCFA production in the intestines are dynamic and are influenced by factors such as the amount and type of fermentation substrate, substrate utilization, and the physiological state of the intestinal flora and host (David et al., 2014). Numerous studies have demonstrated that a diet rich in fiber directly promotes the proliferation of SCFA-producing microorganisms and increases the concentration of SCFAs (De Filippo et al., 2010; Gutiérrez-Díaz et al., 2016; Ho et al., 2016). For example, dietary fiber-rich oats can stimulate the growth and survival of SCFA-producing bacteria such as *Lactobacillus* and Bifidobacterium (Ho et al., 2016). In a cross-sectional study involving a small sample of 31 healthy individuals and focusing on diet structure, it was discovered that levels of Bifidobacterium and Faecalibacterium flora, along with propionate and butyrate concentrations, were significantly higher in the stools of individuals who had adhered to a 6-month Mediterranean diet (a fiber-rich diet) compared to those with lower fiber intake (Gutiérrez-Díaz et al., 2016). This suggests that a fiber-rich diet has a positive impact on SCFA production.

Mechanistically, SCFA is involved in the regulation of host health or disease by binding to cell membrane receptors to trigger intracellular signaling cascades or regulating the epigenetics of intestinal and other tissue cells. Firstly, SCFAs can bind multiple receptors, including free fatty acid receptor 3 [FFAR3 or G protein-coupled receptor 41 (GPR41)], FFAR2 (GPR43), GPR109A (also known as hydroxycarboxylic acid receptor 2 or HCAR2) and olfactory receptor-78 (mouse Olfr78 or human OR51E2) (Martin-Gallausiaux et al., 2021; Lymperopoulos et al., 2022), where FFAR3, FFAR2, and GPR109A are expressed in different organs and cells, such as small intestine, colon, and liver, etc (Dalile et al., 2019). SCFAs activate heterotrimeric G proteins by binding to GPRs, resulting in the separation of the G α subunit from the G βγ subunit. This, in turn, initiates various downstream signaling pathways, such as adenylyl cyclase (AC) and inositol trisphosphate (IP3), and activates key signaling molecules like ERK/Atf2 and mTOR. Consequently, these mechanisms regulate physiological activities such as chemotaxis, apoptosis, proliferation, and differentiation (Flock et al., 2017; Kimura et al., 2020). Olfr78 is mainly expressed in vascular smooth muscle cells of the peripheral vascular system and small renal arteries, where it mediates renin secretion in response to SCFA (Pluznick et al., 2013; Pluznick, 2014; Koh et al., 2016; Pluznick, 2016). Secondly, SCFAs are capable of regulating transcriptional and post-translational modifications by increasing histone acetylation through the inhibition of chromatin histone deacetylase (HDAC) activity. This process promotes the dissociation of DNA from histone octamers and the relaxation of nucleosome structure, facilitating various transcription factors and co-transcription factors to specifically bind to DNA binding sites and activate gene transcription (Shakespear et al., 2011). Additionally, SCFAs act as ligands for two transcription factors, peroxisome proliferator-activated receptor γ (PPARγ) and aryl hydrocarbon receptor (AHR). Through binding to these two transcription factors, SCFAs influence the expression of various genes, including NF-κB and NADPH oxidase (NOX), at the transcriptional level (den Besten et al., 2015; Jin et al., 2017).

**FIGURE 1:**
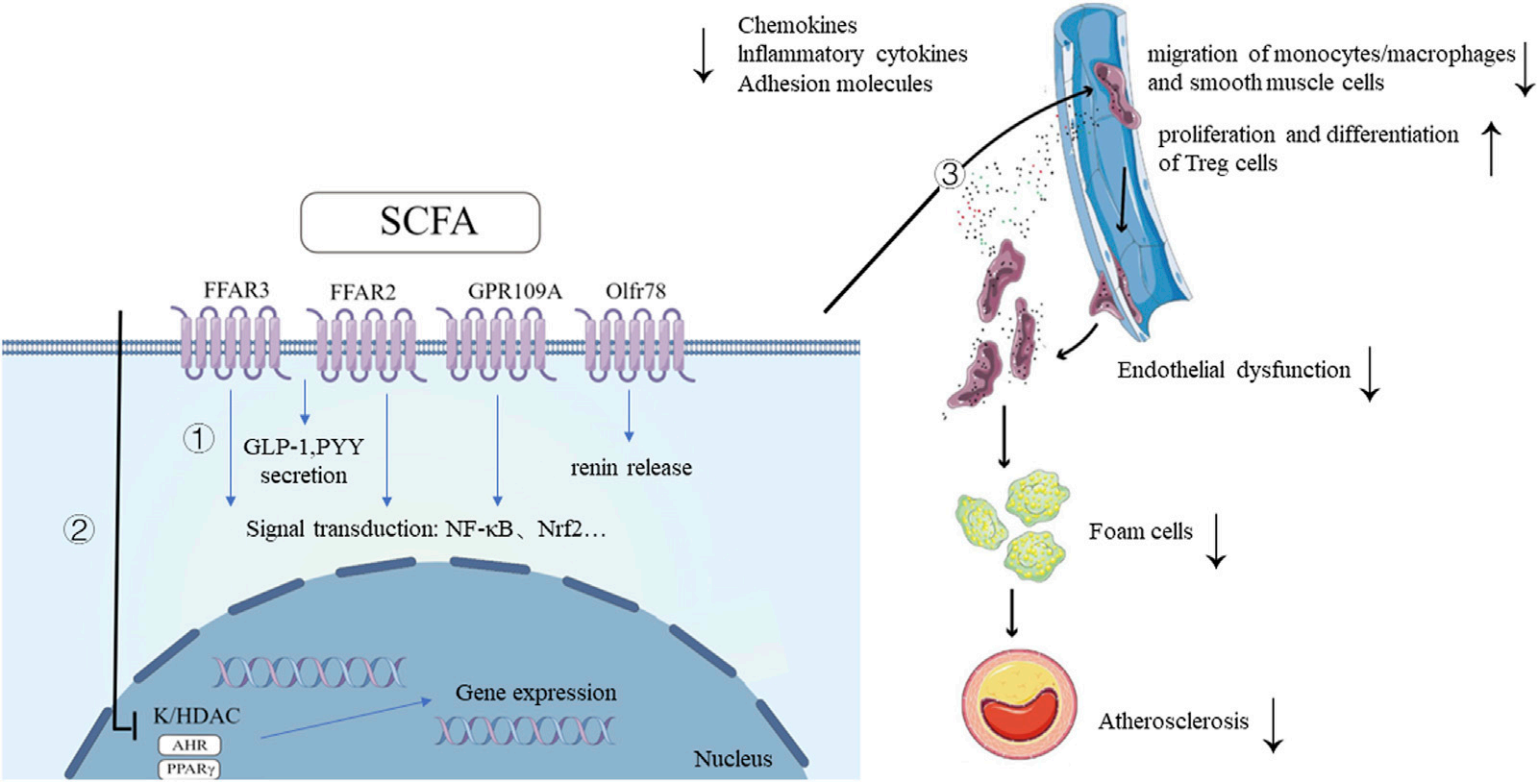
Mechanisms of SCFA and its ameliorative effects in the development of atherosclerosis. 1. SCFAs bind to G protein-coupled receptors, triggering intracellular signaling cascades, leading to downstream signal transduction activation and the secretion of GLP-1, PYY. SCFA also promotes renin secretion by binding to the receptor Olfr78. 2. SCFAs inhibit HDAC activity, promoting histone acetylation, facilitating the dissociation of DNA from histone octamers and relaxation of chromatin structure. This allows various transcription factors and co-transcription factors to bind specifically to DNA binding sites, activating gene transcription. As ligands for AHR and PPARγ, SCFAs regulate the expression of multiple genes at the transcriptional level through their interaction with these two transcription factors. 3. SCFAs improve atherosclerosis by improving inflammation and oxidative stress of endothelial cells, inhibiting migration of monocytes/macrophages, regulating lipid metabolism of macrophages, regulating proliferation and migration of smooth muscle cells and promoting proliferation and differentiation of Treg cells. SCFAs, short-chain fatty acids; FFAR, free fatty acid receptor; GPR109a, G-protein coupled receptor-109a; Olfr78, olfactory receptor-78; AHR, aryl hydrocarbon receptor; HDAC, histone deacetylase; GLP-1, glucagon-like peptide-1; PYY, peptide YY; NF-κB, Nuclear Factor kappa B; Nrf2, NF-E2-related factor 2; TF, transcription factor. Created with FigDraw (www.figdraw.com).

Due to these mechanisms of action (as shown in Figure 1), SCFAs play beneficial roles in human health, such as regulating blood pressure, improving glucolipid metabolism, and modulating immune system. There is growing evidence that SCFAs play an important protective role in the development of atherosclerosis (as shown in Table 1).

**TABLE 1:** The effects and mechanisms of SCFAs in the risk factors of atherosclerosis.

Risk factor	Effect	Cell types	Mechanism	Reference
dyslipidemia	reduce TC, LDL-C and non-HDL-C	peripheral macrophages; hepatocytes; Treg cells	regulate the expression of several key RCT transporter proteins; inhibit intestinal cholesterol absorption by suppressing NPC1L1 expression	Zhao et al. (2017), Tayyeb et al. (2019), Du et al. (2020), Tayyeb et al. (2020), Tayyeb et al. (2021), Haghikia et al. (2022)
hypertension	reduce blood pressure	smooth muscle cells; juxtaglomerular cells	downregulate the renin-angiotensin system; induce vasodilation via Olfr78 and FFAR3; influence the vagal nerve transmission	Lal et al. (2001), Pluznick et al. (2013), Natarajan et al. (2016), Li et al., 2017; Pluznick (2017), Yan et al. (2017), Kim et al. (2018), Huart et al. (2019), Sun et al. (2019), Calderón-Pérez et al. (2020), Verhaar et al. (2020), Liu et al. (2021)
diabetes	improve glucose metabolism	L cells; adipocytes; hepatocytes	promote the release of PYY, GLP-1 and leptin; promote the expression of GLUTs	Xiong et al. (2004), Tolhurst et al. (2012), Psichas et al. (2015), Mollica et al. (2017), Zhao et al. (2018), Medina-Vera et al. (2019), Li et al. (2020), Letchumanan et al. (2022)

TC, total cholesterol; LDL-C, low-density lipoprotein cholesterol; non-HDL-C, non-high-density lipoprotein cholesterol; RCT, reverse cholesterol transport; NPC1L1, Niemann-Pick C1-like protein 1; FFAR, free fatty acid receptor; Olfr78, olfactory receptor-78; PYY, peptide YY; GLP-1, glucagon-like peptide-1; GLUT, glucose transporter protein.

## 3 SCFAs and atherosclerosis with its risk factors

### 3.1 SCFAs and atherosclerosis

In adults, the gastrointestinal tract harbors approximately 100 trillion microorganisms, and many of the SCFA-producing bacteria have been identified through metagenomics and 16S rRNA sequencing (Jia et al., 2008; Louis et al., 2010; Fu et al., 2019). In 2017, the world’s first large-scale atherosclerotic cardiovascular disease (ASCVD) human microbiome study sequenced the gut microbiota composition of 218 ASCVD patients. They found that SCFA-producing bacteria (including Roseburia intestinalis and Faecalibacterium prausnitzii) were less abundant in ASCVD patients compared to healthy controls, suggesting a potential protective role of SCFAs in atherosclerosis (Jie et al., 2017). The reduction in SCFA-producing bacterial abundance in AS patients’ intestines has also been confirmed by other cross-sectional studies (Karlsson et al., 2012; Liu et al., 2019; Zeng et al., 2019).

In animal models, multiple studies have demonstrated the beneficial effects of feeding SCFA preparations in improving atherosclerosis (Aguilar et al., 2014; Kasahara et al., 2018; Bartolomaeus et al., 2019; Haghikia et al., 2022). For example, Aguilar et al. (Aguilar et al., 2014)found that ApoE^−/−^ mice fed with exogenous butyrate experienced a reduction of over 50% in aortic atherosclerotic plaques, with increased plaque stability: a decrease in macrophage numbers, elevated collagen levels, and reduced inflammatory markers. Haghikia et al. (Haghikia et al., 2022) and Bartolomaeus et al. (Bartolomaeus et al., 2019) observed a significant reduction in atherosclerotic lesion areas in ApoE^−/−^ mice treated with propionate, accompanied by a reduction in systemic inflammation. However, there is currently no clinical evidence to definitively establish the outcomes of using SCFA preparations in the treatment of atherosclerosis in human populations, and further research is warranted to elucidate the role of SCFAs in individuals with atherosclerosis.

### 3.2 SCFAs and dyslipidemia

Abnormal blood lipid metabolism is the most important risk factor for atherosclerosis and coronary heart disease. Multiple studies have confirmed that SCFAs can reduce total cholesterol (TC) and low-density lipoprotein cholesterol (LDL-C) levels in the body. For instance, Zhao et al. (2017) fed male hamsters with acetate, propionate, and butyrate for 6 weeks and found that they reduced plasma total cholesterol by 14%, 18%, and 17%, respectively, as well as decreased non-high-density lipoprotein (non-HDL) cholesterol and non-HDL cholesterol/HDL cholesterol ratio. Haghikia et al. (2022) observed significant effects of propionate treatment on lowering TC, VLDL, and LDL-C and preventing the development of atherosclerosis in hypercholesterolemic ApoE^−/−^ mice, further confirming the role of SCFAs in cholesterol regulation. Subsequently, a randomized controlled trial confirmed the lipid-lowering effect of propionate in humans. They found that after 8 weeks of oral administration of 500 mg Bid propionate to individuals with hyperlipidemia, various blood lipid components showed significant reductions: TC decreased by 7.3%, LDL-C decreased by 8.1%, and non-HDL cholesterol decreased by 9.1%, compared to respective reductions of 1.7%, 0.5%, and 0.5% in the control group, indicating that SCFAs are effective cholesterol-lowering agents.

The mechanism by which SCFAs lower circulating cholesterol *in vivo* is related to the regulation of cholesterol transport and excretion by SCFAs. Reverse cholesterol transport (RCT) is a critical process in lipid metabolism, as it transports excess cholesterol from peripheral macrophages to the liver for metabolism and eventual excretion in the feces. To elucidate the potential role of SCFAs in RCT, several animal studies have identified the expression of several key RCT transporter proteins. It was found that butyrate, in particular, upregulated the expression of several genes, including sterol regulatory element binding protein 2 (SREBP2), low-density lipoprotein receptor (LDLR), and cholesterol 7α-hydroxylase (CYP7A1) genes (Tayyeb et al., 2019; Du et al., 2020). Increased hepatic expression of SREBP2 and LDLR promoted the hepatic uptake of circulating cholesterol, while increased CYP7A1 expression reduced blood cholesterol levels by enhancing the excretion of bile acids in feces (Zhao et al., 2017). Both of these conditions contribute to the reduction of circulating cholesterol levels.

Furthermore, Apolipoprotein A-I (ApoA-I) is the major protein in high-density lipoprotein (HDL) and also plays an important role in the process of RCT (Prosser et al., 2012). Tayyeb et al. (2020) found that butyrate inhibited the inflammation-induced decrease in ApoA-I transcription and promoted ApoA-I transcription in hepatic HepG2 cells. Even in a more sophisticated experimental model using transwells, when intestinal Caco-2 cells were co-cultured with HepG2 cells, adding butyrate to the apical side of Caco-2 cells did increase ApoA-I transcription in the HepG2 cells that were cultured in the basolateral compartment (Tayyeb et al., 2021). This suggests that an increase in SCFA flux from the portal vein to the liver may lead to an increase in hepatic ApoA-I transcription.

In addition to hepatic cholesterol metabolism, intestinal cholesterol metabolism is also a key component of cholesterol homeostasis, where Niemann-Pick C1-like protein 1 (NPC1L1) is the major transmembrane transporter protein responsible for intestinal cholesterol absorption (Michos et al., 2019). A recent study found that propionic acid inhibited NPC1L1 expression by increasing the levels of Treg cells and IL-10 in the intestine after feeding propionic acid to hypercholesterolemic ApoE^−/−^ mice, which in turn prevented the increase of TC and LDL-C induced by high-fat diet. This reveals the promising role of SCFAs in improving intestinal cholesterol metabolism by modulating the intestinal immune system (Haghikia et al., 2022).

### 3.3 SCFAs and hypertension

Hypertension is one of the major risk factors for atherosclerosis. There is growing evidence that the abundance of SCFA-producing flora and serum SCFA levels in the gut of hypertensive patients are lower than in the normal population (Li et al., 2017; Yan et al., 2017; Kim et al., 2018; Sun et al., 2019; Calderón-Pérez et al., 2020). For example, a small sample found that SCFA-producing genera (such as Prevotella, Blautia, Coprococcus, Anaerostipes and Ruminococcus) were fewer in the gut of patients with essential hypertension compared to healthy controls (Liu et al., 2021). Another large study including 4,672 subjects from six different ethnic groups participating in Healthy Life in an Urban Setting (HELIUS) described the association between altered gut microbiota composition and blood pressure. Notably, species such as Roseburia spp., *Clostridium* spp., and Romboutsia spp. exhibited significant and inverse associations with blood pressure, indicating the potential of SCFA-producing flora to have an antihypertensive effect (Verhaar et al., 2020). However, interestingly, a longitudinal cohort study of 26 hypertensive patients with a mean follow-up of approximately 5 years showed that SCFAs in the stool of hypertensive patients was higher than in the normal population and was significantly associated with an increase in 24-h mean blood pressure (Huart et al., 2019). This may suggest defects in the absorption of intestinal SCFAs into the body circulation in hypertensive patients (de la Cuesta-Zuluaga et al., 2018; Müller et al., 2019).

In animal models, the role of SCFAs in reducing blood pressure in mice has been confirmed (Bartolomaeus et al., 2019). Several studies have analyzed the roles of SCFA receptors, Olfr78 and FFAR3, in blood pressure regulation, and the results appear to be contradictory. For instance, compared to wild-type mice, Olfr78-deficient mice show a decrease in blood pressure (Pluznick, 2017), while FFAR3 knockout (KO) mice exhibit a hypertensive phenotype (Natarajan et al., 2016). These findings suggest that SCFA receptors Olfr78 and FFAR3 may play antagonistic roles in the regulation of blood pressure. Olfr78 is primarily expressed in the vascular smooth muscle cells of the peripheral vascular system and afferent arterioles of the kidney, and it promotes renin secretion by juxtaglomerular cells. Pluznick et al. (2013) reported that Olfr78 KO mice showed lower blood pressure, which was consistent with the observed lower plasma renin levels in these mice. This points to the importance of SCFA receptor Olfr78-mediated renin secretion in blood pressure regulation. FFAR3 is also expressed in the smooth muscle cells of large arteries and the kidney. Pluznick et al. found that the blood pressure-lowering effect of propionic acid was abolished in FFAR3-deficient mice, suggesting a direct association between its blood pressure-lowering effect and FFAR3 activity. Importantly, FFAR3 has a higher ligand affinity for propionic acid than Olfr78, which may explain the reason for SCFAs’ blood pressure-lowering effect in mice (Le Poul et al., 2003). In summary, Olfr78-mediated activation by propionic acid leads to an increase in blood pressure, while FFAR3-mediated activation results in blood pressure reduction. Olfr78 and FFAR3 play antagonistic roles in blood pressure regulation, balancing each other to achieve healthier blood pressure levels.

Additionally, the influence of SCFAs on blood pressure regulation is also thought to be linked to the gut-brain axis. The activation of SCFA receptors influences the vagal nerve transmission, providing another pathway for the blood pressure-regulating effects of SCFAs (Lal et al., 2001). Animal studies have shown that increased levels of colonic butyrate can lead to blood pressure reduction through activation of the parasympathetic nervous system. When the vagal nerve is severed, the blood pressure-lowering effect of butyrate in rats is significantly diminished, indicating that SCFAs regulate blood pressure through the colonic neural signaling pathway (Onyszkiewicz et al., 2019).

### 3.4 SCFAs and diabetes

Diabetes is closely intertwined with atherosclerosis, and controlling blood glucose is a fundamental measure in preventing and managing atherosclerosis. Research has revealed that various communities of SCFA-producing microbes, including Bifidobacterium, Akkermansia, and Faecalibacterium, are notably reduced in the gut of individuals with prediabetes and type 2 diabetes mellitus (T2DM) when compared to healthy individuals (Li et al., 2020; Letchumanan et al., 2022). This indicates that T2DM patients possess a distinctive gut microbiota composition. Furthermore, several studies have confirmed the role of SCFA-producing microbial communities in blood glucose regulation (Zhao et al., 2018; Medina-Vera et al., 2019). For instance, in a randomized controlled trial involving 81 T2DM patients, Medina-Vera et al. (Medina-Vera et al., 2019) found that after 3 months of a fiber-rich diet, T2DM patients experienced a significant increase in SCFA-producing gut microbial communities (e.g., F. prausnitzii increased by 34%, A. muciniphil increased by 125%), and a noteworthy 7.2% reduction in hemoglobin A1c (HbA1c) levels. Similarly, in another randomized trial involving 43 T2DM patients, Zhao et al. (2018) observed a significant increase in SCFA-producing gut microbial communities after 12 weeks of a fiber-rich diet, leading to approximately 20% improvement in glucose tolerance and a reduction in HbA1c levels. These findings further underscore the role of SCFA-producing microbial communities in blood glucose control within population.

As is well known, blood glucose concentration is regulated by complex hormones, mainly insulin secreted by pancreatic β-cells and glucagon secreted by pancreatic α-cells. Tolhurst et al. (2012) observed that SCFA activation stimulates the secretion of glucagon-like peptide-1 (GLP-1) in L cells (a significant class of enteroendocrine cells) of wild-type mice. However, this stimulatory effect is significantly reduced in FFAR2^−/−^ and FFAR3^−/−^ cells, suggesting the important role of SCFA receptors FFAR2 and FFAR3 in regulating hormone control of blood glucose. Interestingly, *in vivo*, only FFAR2^−/−^ mice showed a significant reduction in circulating GLP-1 levels, while FFAR3^−/−^ mice showed no such effect. It was found that FFAR2 triggers intracellular calcium responses in L cells through specific coupling with Gq protein, thereby enhancing the secretion of GLP-1 (Psichas et al., 2015). Therefore, the attenuated response to SCFAs observed in FFAR3^−/−^ colonic cultures may be related to the decreased expression of FFAR2 observed in FFAR3^−/−^ mice. Furthermore, a recent study found that the SCFA propionic acid stimulates the secretion of the anorectic gut hormones peptide YY (PYY) and GLP-1 in primary mouse colonic organoids from wild-type mice, and this effect is significantly reduced in FFAR2^−/−^ organoids. In an *in vivo* model, the administration of propionic acid in the colon stimulates the release of GLP-1 and PYY in rodents, and this effect was absent in FFAR2^−/−^ mice, once again emphasizing the crucial role of SCFAs in promoting the release of PYY and GLP-1 through FFAR2 (Psichas et al., 2015).

Furthermore, leptin also plays a significant role in blood glucose homeostasis. Research indicates that the SCFA receptor FFAR3 is expressed in adipocytes. SCFAs stimulate the expression of leptin in primary cultures of mouse adipocytes and in mouse adipose tissue through FFAR3 (Xiong et al., 2004). *In vivo* studies have also shown that increasing circulating levels of propionic acid can increase leptin levels in mice, highlighting the role of SCFAs in promoting leptin release (Xiong et al., 2004). Leptin acts on gamma-aminobutyric acid (GABA) and pro-opiomelanocortin (POMC) neurons in the hypothalamus, promoting glucose uptake in brown adipose tissue and skeletal muscles, and improving liver metabolism (Sahu, 2003; Fujikawa et al., 2013). Leptin engages the mentioned hypothalamic neurocircuitry to maintain euglycemia and permit survival in the absence of insulin. Furthermore, leptin directly stimulates liver glycogen synthesis and muscle glucose uptake, thereby lowering blood glucose levels (Minokoshi et al., 2002).

In addition to their impact on hormones, SCFAs also modulate blood glucose by promoting the expression of glucose transporter proteins (GLUTs). GLUTs are a family of transmembrane proteins that regulate the entry of extracellular glucose into cells and are involved in the process of glucose metabolism. It has been reported that after treatment with butyrate, there is an increase in glucose transporter proteins, including GLUT2 and GLUT4, facilitating the cellular uptake of glucose (Mollica et al., 2017).

## 4 The potential mechanisms of SCFAs in improving atherosclerosis

### 4.1 SCFAs improve endothelial cell inflammation and oxidative stress

In the occurrence and development of atherosclerosis, inflammation and oxidative stress play crucial roles in inducing endothelial dysfunction. SCFAs have been discovered to play a role in regulating various inflammatory and oxidative stress pathways, such as NF-κB and Nrf, among others. They mitigate endothelial cell damage, thereby contributing to the improvement of atherosclerosis.

**NF-κB** The NF-κB protein is typically formed by the p65 and p50 subunits, which can form homodimers or heterodimers. In the cytoplasm, NF-κB is in an inactive state due to its binding to the inhibitory protein IκB, forming a trimeric complex. Oxidized low-density lipoprotein (ox-LDL) and pro-inflammatory factors can stimulate the phosphorylation of IκB and the formation of nuclear p65/p50 heterodimers, thereby activating NF-κB and causing endothelial inflammation (Yamamoto and Gaynor, 2001; Yang et al., 2012). SCFAs have been demonstrated to inhibit the expression of various pro-inflammatory genes by suppressing NF-κB activation, thus preventing endothelial leakage. Aguilar et al. (2014) found that pretreatment of EA.hy926 endothelial cells with butyrate effectively inhibited the expression levels of inflammatory factors such as TNF-α, IL-6, and IL-1β. Further mechanistic studies revealed that butyrate reduced the concentration of p65 in endothelial cells and its translocation into the cell nucleus, thereby inhibiting the formation of p65/p50 heterodimers. At the same time, it also increased the concentration of p50 in the cytoplasm. This elevation of cytoplasmic p50 is beneficial for inhibiting the formation of p50/p50 homodimers, thereby reducing the activation of NF-κB genes. Moreover, it has been found that the transcriptional activity of NF-κB can be regulated by the acetylation and deacetylation of proteins in the NF-κB pathway and the accessibility of NF-κB target genes. For example, the subunits of NF-κB (p65 and p50) interact with HDAC to inhibit transcription (Ashburner et al., 2001). Although SCFAs are known as HDAC inhibitors, there is currently no evidence to definitively establish whether their inhibitory effect on NF-κB activity is mediated by HDAC inhibition. Further in-depth research is required in this regard.

**Nrf2** In addition to NF-κB, research has found that SCFAs can promote the expression of Nrf2 and improve endothelial inflammation and oxidative stress. In a Nrf2 knockout diabetic mouse model, Wu et al. (2018) observed that 5% butyrate significantly reduced the expression levels of multiple adhesion molecules and reactive oxygen species components, including 3-nitrotyrosine (3-NT), Vascular Cell Adhesion Molecule-1 (VCAM-1), Intercellular Adhesion Molecule-1 (ICAM-1), and inducible Nitric Oxide Synthase (iNOS) in wild-type mice, by more than 50%. However, this effect was abolished in Nrf2 knockout mice, confirming the involvement of Nrf2 in SCFA-mediated regulation of endothelial inflammation and oxidative stress processes. This effect is primarily exerted by inhibiting HDAC activity and promoting the recruitment of the transcription factor AHR and coactivator P300 to the Nrf2 gene promoter, thereby mediating the activation of Nrf2 transcriptional levels. Moreover, another cell experiment found that after pretreatment with 1 μM propionic acid for 24 h, Nrf2 significantly translocated from the cytoplasm to the nucleus in hCMEC/D3 cells, where it bound to antioxidant response elements (ARE) in the nucleus, leading to a reduction in intracellular ROS levels (Hoyles et al., 2018). The above research indicates that SCFAs activate the Nrf2 signaling pathway through various mechanisms to reduce inflammation and maintain endothelial cell redox homeostasis.

**NOX** NADPH oxidase (NOX) is a critical enzyme involved in intracellular redox signaling, and various NOX isoforms expressed in endothelial cells, such as NOX2 and NOX4, have been shown to increase the production of superoxide, leading to endothelial oxidative stress and the development of atherosclerosis. In an *in vitro* experiment using ox-LDL to stimulate endothelial cells, pretreatment with butyrate (0.5 mM) resulted in a reduction of approximately 70% in the expression of the NOX2 subunit p22phox protein compared to the control group. This effect was achieved through the PPARδ/miR-181b pathway, which downregulates endothelial NOX2 expression and ROS production (Tian et al., 2021).

Additionally, NOX-4 is another target for the treatment of atherosclerosis. While the normal production of NOX-4 is essential for physiological functions, excessive production of NOX-4 can lead to the accumulation of atherosclerotic plaques (Sorescu et al., 2002). In a mouse model study in 2018, it was found that the inhibition of NOX-4 could improve inflammation under conditions of atherosclerosis. Subsequently, in a study by Wang et al. (2020), when examining the effects of butyrate treatment on TNF-α-induced NOX-4 production, it was observed that in the presence of TNF-α alone, endothelial cell NOX-4 expression increased nearly 4-fold at the mRNA level and 3.5-fold at the protein level. However, treatment with 100 μM butyrate reduced the NOX-4 mRNA and protein levels to about 2.5-fold, confirming the effective inhibition of TNF-α-induced overproduction of NOX-4 at both mRNA and protein levels by butyrate. This study demonstrated the value of butyrate in preventing NOX-4-induced atherosclerosis.

### 4.2 SCFAs inhibit migration of monocytes/macrophages

After vascular endothelial injury, monocytes in the bloodstream migrate through the endothelial gap into the subendothelial space, which is a critical step in the development of atherosclerosis (Randolph, 2009). This process is primarily mediated by adhesion molecules, chemokines, and proteases that facilitate monocyte contact, adhesion, and migration with the endothelium. Aguilar et al. (2014) found that in aortic atherosclerosis, butyrate reduced the production of protease MMP2, chemokine CCL2, and adhesion molecule VCAM-1 at the site of lesions, resulting in reduced monocyte migration and adhesion to the lesion area. *In vitro* studies also confirmed that pretreatment with SCFAs inhibited the production of IL-6 and IL-8 and reduced VCAM-1 expression and cell adhesion in human umbilical vein endothelial cells (HUVECs) (Zapolska-Downar et al., 2004; Li et al., 2018). These findings indicate that SCFAs suppress monocyte chemotaxis and adhesion to damaged vascular endothelium by reducing inflammation at the lesion site.

Moreover, the iNOS/Src/FAK axis plays a central role in Toll-like receptor (TLR)-mediated macrophage motility (Maa et al., 2011), where Src (a non-receptor tyrosine kinase) and focal adhesion kinase (FAK) are important proteins associated with cell adhesion and migration (Schlaepfer et al., 1999). In Maa’s study, butyrate was observed to inhibit LPS-induced migration of RAW264.7 macrophages and rat peritoneal macrophages, consistent with the role of iNOS in promoting macrophage mobilization via Src upregulation. After LPS exposure, butyrate pretreatment led to a reduction in iNOS and Src levels, as well as the inhibition of Src and FAK activity in macrophages, indicating that butyrate suppresses LPS-induced macrophage motility by inhibiting the iNOS/Src/FAK axis and reducing their migration to the lesion site (Maa et al., 2010). Furthermore, due to butyrate’s reduction of iNOS, NOX, and ROS production, preventing activated macrophages’ burst of free radicals, it may also facilitate their migration out of the lesion (Steinberg, 2002; Park et al., 2009; Aguilar et al., 2016).

### 4.3 SCFAs regulate lipid metabolism of macrophages

Macrophages phagocytose extracellular lipids through binding with scavenger receptor CD36 and scavenger receptor A (SRA). Excess cholesterol esters within macrophages are broken down within lysosomes and subsequently removed from the cell through the action of transporters known as ATP binding cassette transporter A1 (ABCA-1) and ATP Binding Cassette Subfamily G Member 1 (ABCG-1). This process effectively lowers the levels of cholesterol inside the cell (Platt et al., 2002; Chistiakov et al., 2016; Tian et al., 2020). Zhang et al. (Zhang et al., 2021) found that supplementing high-fat diet rats with FLJ (SCFA supplement) significantly reduced hepatic CD36 mRNA levels by 50%. This was validated *in vitro*, where butyrate (0.5 mM) pretreatment of macrophages resulted in reduced CD36 expression upon ox-LDL stimulation, leading to decreased ox-LDL uptake and intracellular lipid accumulation (Aguilar et al., 2014). Additionally, Du et al. (2020) observed a concentration-dependent upregulation of transporter ABCA-1 expression in macrophages after butyrate pretreatment, with an increase in cholesterol efflux. This effect might be mediated by butyrate’s upregulation of the PI3K pathway, inducing Sp1 phosphorylation, which recruits activated dimers to the ABCA-1 promoter and enhances ABCA-1 transcription (Thymiakou et al., 2007; Chen et al., 2011). However, some studies suggest that butyrate’s influence on macrophage ABCA-1 may also be mediated by SCFA receptor GPR109A, as its activation stimulates ABCA-1 expression in macrophages, subsequently promoting cholesterol efflux mediated by ApoA-I (Chai et al., 2013; Gaidarov et al., 2013; Tumurkhuu et al., 2018). These mechanisms activate ABCA-1 expression and may promote cholesterol efflux, reducing foam cell formation.

### 4.4 SCFAs regulate proliferation and migration of smooth muscle cells

The structural and functional changes in vascular smooth muscle cells (VSMCs) are closely associated with the development of atherosclerosis, promoting plaque formation and inducing plaque instability. Studies have found that butyrate can significantly inhibit VSMC proliferation, improve arterial wall thickness, and reduce collagen deposition (Tan et al., 2017).

The protective effects of butyrate on atherosclerosis are achieved through the regulation of VSMC histone epigenetic modifications, appropriately altering chromatin dynamics, and thereby modulating the expression of G1-specific cell cycle proteins to halt VSMC proliferation. For example, Mathew et al. (2010) found that the interaction between different site-specific post-translational modifications of histone H3 in butyrate-treated VSMCs seems to alter chromatin structure and organization, resulting in a stalling of VSMC proliferation by regulating differential expression of cell cycle negative regulators and positive regulators. Specifically, the collaborative effects of butyrate on histone H3 Lys9 acetylation and H3 Ser10 phosphorylation, as well as the contrasting effects on H3 Lys4 and H3 Lys9 dimethylation, lead to downregulation of D-type cyclins (cyclin D1 and D3) and G1-specific cyclin-dependent kinases (cdk2, cdk4, and cdk6), and upregulation of cdk inhibitors p15INK4b and p21Cip1 (Milton et al., 2012). The downregulation of cdk2, cdk4, and cdk6 and upregulation of p15INK4b and p21Cip1 are consistent with butyrate’s anti-proliferative effects, confirming that butyrate inhibits VSMC proliferation by regulating their epigenetics.

Furthermore, butyrate increases the expression of glutathione peroxidase (GPX) 3 and GPX4, enhancing the overall catalytic activity of the GPX family to promote antioxidant effects and inhibit VSMC proliferation (Mathew et al., 2010; Mathew et al., 2014). Another study suggested that butyrate’s anti-proliferative effect on VSMCs is achieved through its impact on HDAC activity and phosphoinositide 3-kinase/Akt pathway, involving inhibition of protein synthesis and apoptosis (Mathew et al., 2019). Lastly, butyrate can inhibit platelet-derived growth factor (PDGF)-induced migration of pulmonary artery VSMCs, possibly through downregulation of PDGF receptor transcription and inhibition of PDGF-induced Akt phosphorylation (Cantoni et al., 2013).

### 4.5 SCFAs promote proliferation and differentiation of Treg cells

Atherosclerosis plaques are enriched with lymphocytes, and regulatory T cells (Tregs) have been a focus of research in recent years for their protective role in AS. Studies have shown that SCFAs can regulate adaptive immune responses by directly or indirectly modulating Treg cell differentiation and proliferation. A recent study published in the “European Heart Journal” found that in subjects with hyperlipidemia, plasma propionate concentration significantly increased after 8 weeks of oral supplementation with propionate, leading to a 5.5% increase in peripheral Treg cells. Importantly, the numbers of Th17 cells or Th1 cells did not exhibit significant changes (Haghikia et al., 2022). This is consistent with the results observed by Duscha et al. (2020) in patients with multiple sclerosis (MS), where Treg cell numbers increased by 30% after 14 days of propionate supplementation in MS patients and by approximately 25% in healthy controls, suggesting the role of SCFAs in promoting Treg cell proliferation. Furthermore, Bartolomaeus et al. (2019) found that propionate significantly reduced systemic inflammatory responses, markedly lowered susceptibility to ventricular arrhythmias, and reduced atherosclerosis and cardiac remodeling in a model of high blood pressure. The protective effects of propionate on target organs partially depended on the proliferation of Tregs, and depletion of Treg cells eliminated the beneficial effects of propionate on systemic and cardiac inflammation and fibrosis. These studies provide strong evidence that SCFAs can improve the inflammatory state by regulating Treg cell proliferation.

Currently, SCFAs have been confirmed as key metabolic products that promote the differentiation of naive T cells into Tregs within the intestinal tract (Atarashi et al., 2011; Arpaia et al., 2013; Atarashi et al., 2013; Furusawa et al., 2013; Smith et al., 2013; Singh et al., 2014). The induction of Tregs by SCFAs is believed to be mediated through the inhibition of HDAC, thereby regulating the epigenetics of Treg cells (Tanoue et al., 2016). For instance, recent studies in mice suggest that butyrate may enhance the acetylation of histone H3 at the Foxp3 locus through HDAC inhibition. This leads to a more relaxed chromatin structure, thereby inducing Foxp3 gene expression (Furusawa et al., 2013). Furthermore, at the molecular level, butyrate has been shown to promote the acetylation of the Foxp3 protein, preventing proteasomal degradation, and enhancing the stability and activity of this transcription factor (Arpaia et al., 2013). The molecular mechanisms underlying the control of Treg development by SCFAs are complex and involve various cell types, such as myeloid cells and intestinal epithelial cells (IECs). Studies have found that butyrate-mediated GPR109A signaling activation promotes macrophages to exhibit anti-inflammatory properties, leading to the promotion of Foxp3+ Tregs and IL-10-producing CD4^+^ T cells in the colon (Atarashi et al., 2013). Additionally, SCFAs can also increase the production of TGF-β1 by inhibiting HDAC activity in IECs, thereby promoting Tregs differentiation (Atarashi et al., 2011; Atarashi et al., 2013; Martin-Gallausiaux et al., 2018).

## 5 Conclusion

Atherosclerosis is a global health problem, and the development of effective treatment plans is of paramount importance. SCFAs, as one of the metabolites derived from the gut microbiota, have garnered significant attention as a potential therapeutic approach for atherosclerosis. However, the mechanisms by which SCFAs alleviate atherosclerosis are still in their early stages. In this review, we elucidate the research progress on SCFAs regarding their impact on the risk factors and pathogenesis associated with atherosclerosis, with a specific focus on their interactions with the endothelium and immune cells. It is evident that the beneficial properties of SCFAs hold the potential for preventing and treating atherosclerosis. Nevertheless, several issues need to be addressed before the clinical application of SCFAs.

First, most of these SCFA-induced beneficial results were observed in animal experiments and have rarely been performed in humans. Further research should be conducted to confirm whether these benefits could be repeated in humans. Second, the current body of research is insufficient to comprehensively understand the full spectrum of SCFAs’ mechanisms of action. Therefore, further in-depth investigations are imperative to establish a solid theoretical foundation for the development of clinical therapeutics in this context.

We are confident that these difficulties will and must be overcome to elucidate the detailed mechanism of the function of SCFAs and their promising medical value to mankind.

## References

[B1] Aguilar E. C. Leonel A. J. Teixeira L. G. Silva A. R. Silva J. F. Pelaez J. M. N. (2014). Butyrate impairs atherogenesis by reducing plaque inflammation and vulnerability and decreasing NFκB activation. Nutr. Metab. Cardiovasc Dis. 24 (6), 606–613. 10.1016/j.numecd.2014.01.002 24602606

[B2] Aguilar E. C. Santos L. C. D. Leonel A. J. de Oliveira J. S. Santos E. A. Navia-Pelaez J. M. (2016). Oral butyrate reduces oxidative stress in atherosclerotic lesion sites by a mechanism involving NADPH oxidase down-regulation in endothelial cells. J. Nutr. Biochem. 34, 99–105. 10.1016/j.jnutbio.2016.05.002 27261536

[B3] Arpaia N. Campbell C. Fan X. Dikiy S. van der Veeken J. deRoos P. (2013). Metabolites produced by commensal bacteria promote peripheral regulatory T-cell generation. Nature 504 (7480), 451–455. 10.1038/nature12726 24226773 PMC3869884

[B4] Ashburner B. P. Westerheide S. D. Baldwin A. S. Jr. (2001). The p65 (RelA) subunit of NF-kappaB interacts with the histone deacetylase (HDAC) corepressors HDAC1 and HDAC2 to negatively regulate gene expression. Mol. Cell Biol. 21 (20), 7065–7077. 10.1128/MCB.21.20.7065-7077.2001 11564889 PMC99882

[B5] Atarashi K. Tanoue T. Oshima K. Suda W. Nagano Y. Nishikawa H. (2013). Treg induction by a rationally selected mixture of Clostridia strains from the human microbiota. Nature 500 (7461), 232–236. 10.1038/nature12331 23842501

[B6] Atarashi K. Tanoue T. Shima T. Imaoka A. Kuwahara T. Momose Y. (2011). Induction of colonic regulatory T cells by indigenous Clostridium species. Science 331 (6015), 337–341. 10.1126/science.1198469 21205640 PMC3969237

[B7] Bartolomaeus H. Balogh A. Yakoub M. Homann S. Markó L. Höges S. (2019). Short-chain fatty acid propionate protects from hypertensive cardiovascular damage. Circulation 139 (11), 1407–1421. 10.1161/CIRCULATIONAHA.118.036652 30586752 PMC6416008

[B8] Calderón-Pérez L. Gosalbes M. J. Yuste S. Valls R. M. Pedret A. Llauradó E. (2020). Gut metagenomic and short chain fatty acids signature in hypertension: a cross-sectional study. Sci. Rep. 10 (1), 6436. 10.1038/s41598-020-63475-w 32296109 PMC7160119

[B9] Cani P. D. Amar J. Iglesias M. A. Poggi M. Knauf C. Bastelica D. (2007). Metabolic endotoxemia initiates obesity and insulin resistance. Diabetes 56 (7), 1761–1772. 10.2337/db06-1491 17456850

[B10] Cantoni S. Galletti M. Zambelli F. Valente S. Ponti F. Tassinari R. (2013). Sodium butyrate inhibits platelet-derived growth factor-induced proliferation and migration in pulmonary artery smooth muscle cells through Akt inhibition. Febs J. 280 (9), 2042–2055. 10.1111/febs.12227 23463962

[B11] Chai J. T. Digby J. E. Choudhury R. P. (2013). GPR109A and vascular inflammation. Curr. Atheroscler. Rep. 15 (5), 325. 10.1007/s11883-013-0325-9 23526298 PMC3631117

[B12] Chen X. Zhao Y. Guo Z. Zhou L. Okoro E. U. Yang H. (2011). Transcriptional regulation of ATP-binding cassette transporter A1 expression by a novel signaling pathway. J. Biol. Chem. 286 (11), 8917–8923. 10.1074/jbc.M110.214429 21257755 PMC3058999

[B13] Chistiakov D. A. Bobryshev Y. V. Orekhov A. N. (2016). Macrophage-mediated cholesterol handling in atherosclerosis. J. Cell Mol. Med. 20 (1), 17–28. 10.1111/jcmm.12689 26493158 PMC4717859

[B14] Cummings J. H. (1981). Short chain fatty acids in the human colon. Gut 22 (9), 763–779. 10.1136/gut.22.9.763 7028579 PMC1419865

[B15] Dalile B. Van Oudenhove L. Vervliet B. Verbeke K. (2019). The role of short-chain fatty acids in microbiota-gut-brain communication. Nat. Rev. Gastroenterol. Hepatol. 16 (8), 461–478. 10.1038/s41575-019-0157-3 31123355

[B16] David L. A. Maurice C. F. Carmody R. N. Gootenberg D. B. Button J. E. Wolfe B. E. (2014). Diet rapidly and reproducibly alters the human gut microbiome. Nature 505 (7484), 559–563. 10.1038/nature12820 24336217 PMC3957428

[B17] De Filippo C. Cavalieri D. Di Paola M. Ramazzotti M. Poullet J. B. Massart S. (2010). Impact of diet in shaping gut microbiota revealed by a comparative study in children from Europe and rural Africa. Proc. Natl. Acad. Sci. U. S. A. 107 (33), 14691–14696. 10.1073/pnas.1005963107 20679230 PMC2930426

[B18] de la Cuesta-Zuluaga J. Mueller N. T. Álvarez-Quintero R. Velásquez-Mejía E. P. Sierra J. A. Corrales-Agudelo V. (2018). Higher fecal short-chain fatty acid levels are associated with gut microbiome dysbiosis, obesity, hypertension and cardiometabolic disease risk factors. Nutrients 11 (1), 51. 10.3390/nu11010051 30591685 PMC6356834

[B19] den Besten G. Bleeker A. Gerding A. van Eunen K. Havinga R. van Dijk T. H. (2015). Short-chain fatty acids protect against high-fat diet-induced obesity via a pparγ-dependent switch from lipogenesis to fat oxidation. Diabetes 64 (7), 2398–2408. 10.2337/db14-1213 25695945

[B20] den Besten G. van Eunen K. Groen A. K. Venema K. Reijngoud D. J. Bakker B. M. (2013). The role of short-chain fatty acids in the interplay between diet, gut microbiota, and host energy metabolism. J. Lipid Res. 54 (9), 2325–2340. 10.1194/jlr.R036012 23821742 PMC3735932

[B21] Du Y. Li X. Su C. Xi M. Zhang X. Jiang Z. (2020). Butyrate protects against high-fat diet-induced atherosclerosis via up-regulating ABCA1 expression in apolipoprotein E-deficiency mice. Br. J. Pharmacol. 177 (8), 1754–1772. 10.1111/bph.14933 31769014 PMC7070171

[B22] Duscha A. Gisevius B. Hirschberg S. Yissachar N. Stangl G. I. Eilers E. (2020). Propionic acid shapes the multiple sclerosis disease course by an immunomodulatory mechanism. Cell 180 (6), 1067–1080. 10.1016/j.cell.2020.02.035 32160527

[B23] Flock T. Hauser A. S. Lund N. Gloriam D. E. Balaji S. Babu M. M. (2017). Selectivity determinants of GPCR-G-protein binding. Nature 545 (7654), 317–322. 10.1038/nature22070 28489817 PMC5846738

[B24] Fu X. Liu Z. Zhu C. Mou H. Kong Q. (2019). Nondigestible carbohydrates, butyrate, and butyrate-producing bacteria. Crit. Rev. Food Sci. Nutr. 59 (Suppl. 1), S130–s152. 10.1080/10408398.2018.1542587 30580556

[B25] Fujikawa T. Berglund E. D. Patel V. R. Ramadori G. Vianna C. R. Vong L. (2013). Leptin engages a hypothalamic neurocircuitry to permit survival in the absence of insulin. Cell Metab. 18 (3), 431–444. 10.1016/j.cmet.2013.08.004 24011077 PMC3890693

[B26] Furusawa Y. Obata Y. Fukuda S. Endo T. A. Nakato G. Takahashi D. (2013). Commensal microbe-derived butyrate induces the differentiation of colonic regulatory T cells. Nature 504 (7480), 446–450. 10.1038/nature12721 24226770

[B27] Gaidarov I. Chen X. Anthony T. Maciejewski-Lenoir D. Liaw C. Unett D. J. (2013). Differential tissue and ligand-dependent signaling of GPR109A receptor: implications for anti-atherosclerotic therapeutic potential. Cell Signal 25 (10), 2003–2016. 10.1016/j.cellsig.2013.06.008 23770183

[B28] Gutiérrez-Díaz I. Fernández-Navarro T. Sánchez B. Margolles A. González S. (2016). Mediterranean diet and faecal microbiota: a transversal study. Food Funct. 7 (5), 2347–2356. 10.1039/c6fo00105j 27137178

[B29] Haghikia A. Zimmermann F. Schumann P. Jasina A. Roessler J. Schmidt D. (2022). Propionate attenuates atherosclerosis by immune-dependent regulation of intestinal cholesterol metabolism. Eur. Heart J. 43 (6), 518–533. 10.1093/eurheartj/ehab644 34597388 PMC9097250

[B30] Ho H. V. Sievenpiper J. L. Zurbau A. Blanco Mejia S. Jovanovski E. Au-Yeung F. (2016). The effect of oat β-glucan on LDL-cholesterol, non-HDL-cholesterol and apoB for CVD risk reduction: a systematic review and meta-analysis of randomised-controlled trials. Br. J. Nutr. 116 (8), 1369–1382. 10.1017/S000711451600341X 27724985

[B31] Hooper L. V. Littman D. R. Macpherson A. J. (2012). Interactions between the microbiota and the immune system. Science 336 (6086), 1268–1273. 10.1126/science.1223490 22674334 PMC4420145

[B32] Hoyles L. Snelling T. Umlai U. K. Nicholson J. K. Carding S. R. Glen R. C. (2018). Microbiome-host systems interactions: protective effects of propionate upon the blood-brain barrier. Microbiome 6 (1), 55. 10.1186/s40168-018-0439-y 29562936 PMC5863458

[B33] Hu T. Wu Q. Yao Q. Jiang K. Yu J. Tang Q. (2022). Short-chain fatty acid metabolism and multiple effects on cardiovascular diseases. Ageing Res. Rev. 81, 101706. 10.1016/j.arr.2022.101706 35932976

[B34] Huart J. Leenders J. Taminiau B. Descy J. Saint-Remy A. Daube G. (2019). Gut microbiota and fecal levels of short-chain fatty acids differ upon 24-hour blood pressure levels in men. Hypertension 74 (4), 1005–1013. 10.1161/HYPERTENSIONAHA.118.12588 31352822

[B35] Jandhyala S. M. Talukdar R. Subramanyam C. Vuyyuru H. Sasikala M. Nageshwar Reddy D. (2015). Role of the normal gut microbiota. World J. Gastroenterol. 21 (29), 8787–8803. 10.3748/wjg.v21.i29.8787 26269668 PMC4528021

[B36] Jia W. Li H. Zhao L. Nicholson J. K. (2008). Gut microbiota: a potential new territory for drug targeting. Nat. Rev. Drug Discov. 7 (2), 123–129. 10.1038/nrd2505 18239669

[B37] Jie Z. Xia H. Zhong S. L. Feng Q. Li S. Liang S. (2017). The gut microbiome in atherosclerotic cardiovascular disease. Nat. Commun. 8 (1), 845. 10.1038/s41467-017-00900-1 29018189 PMC5635030

[B38] Jin U. H. Cheng Y. Park H. Davidson L. A. Callaway E. S. Chapkin R. S. (2017). Short chain fatty acids enhance aryl hydrocarbon (ah) responsiveness in mouse colonocytes and caco-2 human colon cancer cells. Sci. Rep. 7 (1), 10163. 10.1038/s41598-017-10824-x 28860561 PMC5579248

[B39] Karlsson F. H. Fåk F. Nookaew I. Tremaroli V. Fagerberg B. Petranovic D. (2012). Symptomatic atherosclerosis is associated with an altered gut metagenome. Nat. Commun. 3, 1245. 10.1038/ncomms2266 23212374 PMC3538954

[B40] Kasahara K. Krautkramer K. A. Org E. Romano K. A. Kerby R. L. Vivas E. I. (2018). Interactions between Roseburia intestinalis and diet modulate atherogenesis in a murine model. Nat. Microbiol. 3 (12), 1461–1471. 10.1038/s41564-018-0272-x 30397344 PMC6280189

[B41] Kim S. Goel R. Kumar A. Qi Y. Lobaton G. Hosaka K. (2018). Imbalance of gut microbiome and intestinal epithelial barrier dysfunction in patients with high blood pressure. Clin. Sci. (Lond) 132 (6), 701–718. 10.1042/CS20180087 29507058 PMC5955695

[B42] Kimura I. Ichimura A. Ohue-Kitano R. Igarashi M. (2020). Free fatty acid receptors in health and disease. Physiol. Rev. 100 (1), 171–210. 10.1152/physrev.00041.2018 31487233

[B43] Koh A. De Vadder F. Kovatcheva-Datchary P. Bäckhed F. (2016). From dietary fiber to host physiology: short-chain fatty acids as key bacterial metabolites. Cell 165 (6), 1332–1345. 10.1016/j.cell.2016.05.041 27259147

[B44] Korem T. Zeevi D. Suez J. Weinberger A. Avnit-Sagi T. Pompan-Lotan M. (2015). Growth dynamics of gut microbiota in health and disease inferred from single metagenomic samples. Science 349 (6252), 1101–1106. 10.1126/science.aac4812 26229116 PMC5087275

[B45] Lal S. Kirkup A. J. Brunsden A. M. Thompson D. G. Grundy D. (2001). Vagal afferent responses to fatty acids of different chain length in the rat. Am. J. Physiol. Gastrointest. Liver Physiol. 281 (4), G907–G915. 10.1152/ajpgi.2001.281.4.G907 11557510

[B46] Le Poul E. Loison C. Struyf S. Springael J. Y. Lannoy V. Decobecq M. E. (2003). Functional characterization of human receptors for short chain fatty acids and their role in polymorphonuclear cell activation. J. Biol. Chem. 278 (28), 25481–25489. 10.1074/jbc.M301403200 12711604

[B47] Letchumanan G. Abdullah N. Marlini M. Baharom N. Lawley B. Omar M. R. (2022). Gut microbiota composition in prediabetes and newly diagnosed type 2 diabetes: a systematic review of observational studies. Front. Cell Infect. Microbiol. 12, 943427. 10.3389/fcimb.2022.943427 36046745 PMC9422273

[B48] Li J. Zhao F. Wang Y. Chen J. Tao J. Tian G. (2017). Gut microbiota dysbiosis contributes to the development of hypertension. Microbiome 5 (1), 14. 10.1186/s40168-016-0222-x 28143587 PMC5286796

[B49] Li M. van Esch B. C. A. M. Henricks P. A. J. Garssen J. Folkerts G. (2018). Time and concentration dependent effects of short chain fatty acids on lipopolysaccharide- or tumor necrosis factor α-induced endothelial activation. Front. Pharmacol. 9, 233. 10.3389/fphar.2018.00233 29615908 PMC5867315

[B50] Li Q. Chang Y. Zhang K. Chen H. Tao S. Zhang Z. (2020). Implication of the gut microbiome composition of type 2 diabetic patients from northern China. Sci. Rep. 10 (1), 5450. 10.1038/s41598-020-62224-3 32214153 PMC7096501

[B51] Liu H. Chen X. Hu X. Niu H. Tian R. Wang H. (2019). Alterations in the gut microbiome and metabolism with coronary artery disease severity. Microbiome 7 (1), 68. 10.1186/s40168-019-0683-9 31027508 PMC6486680

[B52] Liu Y. Jiang Q. Liu Z. Shen S. Ai J. Zhu Y. (2021). Alteration of gut microbiota relates to metabolic disorders in primary aldosteronism patients. Front. Endocrinol. (Lausanne) 12, 667951. 10.3389/fendo.2021.667951 34484110 PMC8415980

[B53] Louis P. Young P. Holtrop G. Flint H. J. (2010). Diversity of human colonic butyrate-producing bacteria revealed by analysis of the butyryl-CoA:acetate CoA-transferase gene. Environ. Microbiol. 12 (2), 304–314. 10.1111/j.1462-2920.2009.02066.x 19807780

[B54] Lu Y. Zhang Y. Zhao X. Shang C. Xiang M. Li L. (2022). Microbiota-derived short-chain fatty acids: implications for cardiovascular and metabolic disease. Front. Cardiovasc Med. 9, 900381. 10.3389/fcvm.2022.900381 36035928 PMC9403138

[B55] Lymperopoulos A. Suster M. S. Borges J. I. (2022). Short-chain fatty acid receptors and cardiovascular function. Int. J. Mol. Sci. 23 (6), 3303. 10.3390/ijms23063303 35328722 PMC8952772

[B56] Maa M. C. Chang M. Y. Hsieh M. Y. Chen Y. J. Yang C. J. Chen Z. C. (2010). Butyrate reduced lipopolysaccharide-mediated macrophage migration by suppression of Src enhancement and focal adhesion kinase activity. J. Nutr. Biochem. 21 (12), 1186–1192. 10.1016/j.jnutbio.2009.10.004 20149623

[B57] Maa M. C. Chang M. Y. Li J. Li Y. Y. Hsieh M. Y. Yang C. J. (2011). The iNOS/Src/FAK axis is critical in Toll-like receptor-mediated cell motility in macrophages. Biochim. Biophys. Acta 1813 (1), 136–147. 10.1016/j.bbamcr.2010.09.004 20849883

[B58] Macfarlane G. T. Macfarlane S. (1997). Human colonic microbiota: ecology, physiology and metabolic potential of intestinal bacteria. Scand. J. Gastroenterol. Suppl. 222, 3–9. 10.1080/00365521.1997.11720708 9145437

[B59] Makki K. Deehan E. C. Walter J. Bäckhed F. (2018). The impact of dietary fiber on gut microbiota in host health and disease. Cell Host Microbe 23 (6), 705–715. 10.1016/j.chom.2018.05.012 29902436

[B60] Martin-Gallausiaux C. Béguet-Crespel F. Marinelli L. Jamet A. Ledue F. Blottière H. M. (2018). Butyrate produced by gut commensal bacteria activates TGF-beta1 expression through the transcription factor SP1 in human intestinal epithelial cells. Sci. Rep. 8(1). 9742. 10.1038/s41598-018-28048-y 29950699 PMC6021401

[B61] Martin-Gallausiaux C. Marinelli L. Blottière H. M. Larraufie P. Lapaque N. (2021). SCFA: mechanisms and functional importance in the gut. Proc. Nutr. Soc. 80 (1), 37–49. 10.1017/S0029665120006916 32238208

[B62] Mathew O. P. Ranganna K. Mathew J. Zhu M. Yousefipour Z. Selvam C. (2019). Cellular effects of butyrate on vascular smooth muscle cells are mediated through disparate actions on dual targets, histone deacetylase (HDAC) activity and PI3K/Akt signaling network. Int. J. Mol. Sci. 20 (12), 2902. 10.3390/ijms20122902 31197106 PMC6628026

[B63] Mathew O. P. Ranganna K. Milton S. G. (2014). Involvement of the antioxidant effect and anti-inflammatory response in butyrate-inhibited vascular smooth muscle cell proliferation. Pharm. (Basel) 7 (11), 1008–1027. 10.3390/ph7111008 PMC4246201 25390157

[B64] Mathew O. P. Ranganna K. Yatsu F. M. (2010). Butyrate, an HDAC inhibitor, stimulates interplay between different posttranslational modifications of histone H3 and differently alters G1-specific cell cycle proteins in vascular smooth muscle cells. Biomed. Pharmacother. 64 (10), 733–740. 10.1016/j.biopha.2010.09.017 20970954 PMC2997917

[B65] Medina-Vera I. Sanchez-Tapia M. Noriega-López L. Granados-Portillo O. Guevara-Cruz M. Flores-López A. (2019). A dietary intervention with functional foods reduces metabolic endotoxaemia and attenuates biochemical abnormalities by modifying faecal microbiota in people with type 2 diabetes. Diabetes Metab. 45 (2), 122–131. 10.1016/j.diabet.2018.09.004 30266575

[B66] Michos E. D. McEvoy J. W. Blumenthal R. S. (2019). Lipid management for the prevention of atherosclerotic cardiovascular disease. N. Engl. J. Med. 381 (16), 1557–1567. 10.1056/NEJMra1806939 31618541

[B67] Milton S. G. Mathew O. P. Yatsu F. M. Ranganna K. (2012). Differential cellular and molecular effects of butyrate and trichostatin a on vascular smooth muscle cells. Pharm. (Basel) 5 (9), 925–943. 10.3390/ph5090925 PMC3816648 24280698

[B68] Minokoshi Y. Kim Y. B. Peroni O. D. Fryer L. G. D. Müller C. Carling D. (2002). Leptin stimulates fatty-acid oxidation by activating AMP-activated protein kinase. Nature 415 (6869), 339–343. 10.1038/415339a 11797013

[B69] Mollica M. P. Mattace Raso G. Cavaliere G. Trinchese G. De Filippo C. Aceto S. (2017). Butyrate regulates liver mitochondrial function, efficiency, and dynamics in insulin-resistant obese mice. Diabetes 66 (5), 1405–1418. 10.2337/db16-0924 28223285

[B70] Müller M. Hernández M. A. G. Goossens G. H. Reijnders D. Holst J. J. Jocken J. W. E. (2019). Circulating but not faecal short-chain fatty acids are related to insulin sensitivity, lipolysis and GLP-1 concentrations in humans. Sci. Rep. 9 (1), 12515. 10.1038/s41598-019-48775-0 31467327 PMC6715624

[B71] Natarajan N. Hori D. Flavahan S. Steppan J. Flavahan N. A. Berkowitz D. E. (2016). Microbial short chain fatty acid metabolites lower blood pressure via endothelial G protein-coupled receptor 41. Physiol. Genomics 48 (11), 826–834. 10.1152/physiolgenomics.00089.2016 27664183 PMC6223570

[B72] Onyszkiewicz M. Gawrys-Kopczynska M. Konopelski P. Aleksandrowicz M. Sawicka A. Koźniewska E. (2019). Butyric acid, a gut bacteria metabolite, lowers arterial blood pressure via colon-vagus nerve signaling and GPR41/43 receptors. Pflugers Arch. 471 (11-12), 1441–1453. 10.1007/s00424-019-02322-y 31728701 PMC6882756

[B73] Park Y. M. Febbraio M. Silverstein R. L. (2009). CD36 modulates migration of mouse and human macrophages in response to oxidized LDL and may contribute to macrophage trapping in the arterial intima. J. Clin. Invest. 119 (1), 136–145. 10.1172/JCI35535 19065049 PMC2613464

[B74] Platt N. Haworth R. Darley L. Gordon S. (2002). The many roles of the class A macrophage scavenger receptor. Int. Rev. Cytol. 212, 1–40. 10.1016/s0074-7696(01)12002-4 11804035

[B75] Pluznick J. (2014). A novel SCFA receptor, the microbiota, and blood pressure regulation. Gut Microbes 5 (2), 202–207. 10.4161/gmic.27492 24429443 PMC4063845

[B76] Pluznick J. L. (2016). Gut microbiota in renal physiology: focus on short-chain fatty acids and their receptors. Kidney Int. 90 (6), 1191–1198. 10.1016/j.kint.2016.06.033 27575555 PMC5123942

[B77] Pluznick J. L. (2017). Microbial short-chain fatty acids and blood pressure regulation. Curr. Hypertens. Rep. 19 (4), 25. 10.1007/s11906-017-0722-5 28315048 PMC5584783

[B78] Pluznick J. L. Protzko R. J. Gevorgyan H. Peterlin Z. Sipos A. Han J. (2013). Olfactory receptor responding to gut microbiota-derived signals plays a role in renin secretion and blood pressure regulation. Proc. Natl. Acad. Sci. U. S. A. 110 (11), 4410–4415. 10.1073/pnas.1215927110 23401498 PMC3600440

[B79] Prosser H. C. Ng M. K. C. Bursill C. A. (2012). The role of cholesterol efflux in mechanisms of endothelial protection by HDL. Curr. Opin. Lipidol. 23 (3), 182–189. 10.1097/MOL.0b013e328352c4dd 22488423

[B80] Psichas A. Sleeth M. L. Murphy K. G. Brooks L. Bewick G. A. Hanyaloglu A. C. (2015). The short chain fatty acid propionate stimulates GLP-1 and PYY secretion via free fatty acid receptor 2 in rodents. Int. J. Obes. (Lond) 39 (3), 424–429. 10.1038/ijo.2014.153 25109781 PMC4356745

[B81] Randolph G. J. (2009). The fate of monocytes in atherosclerosis. J. Thromb. Haemost. 7 (1). 28–30. 10.1111/j.1538-7836.2009.03423.x 19630762 PMC2862012

[B82] Sahu A. (2003). Leptin signaling in the hypothalamus: emphasis on energy homeostasis and leptin resistance. Front. Neuroendocrinol. 24 (4), 225–253. 10.1016/j.yfrne.2003.10.001 14726256

[B83] Schlaepfer D. D. Hauck C. R. Sieg D. J. (1999). Signaling through focal adhesion kinase. Prog. Biophys. Mol. Biol. 71 (3-4), 435–478. 10.1016/s0079-6107(98)00052-2 10354709

[B84] Shakespear M. R. Halili M. A. Irvine K. M. Fairlie D. P. Sweet M. J. (2011). Histone deacetylases as regulators of inflammation and immunity. Trends Immunol. 32 (7), 335–343. 10.1016/j.it.2011.04.001 21570914

[B85] Singh N. Gurav A. Sivaprakasam S. Brady E. Padia R. Shi H. (2014). Activation of Gpr109a, receptor for niacin and the commensal metabolite butyrate, suppresses colonic inflammation and carcinogenesis. Immunity 40 (1), 128–139. 10.1016/j.immuni.2013.12.007 24412617 PMC4305274

[B86] Smith P. M. Howitt M. R. Panikov N. Michaud M. Gallini C. A. Bohlooly-Y M. (2013). The microbial metabolites, short-chain fatty acids, regulate colonic Treg cell homeostasis. Science 341 (6145), 569–573. 10.1126/science.1241165 23828891 PMC3807819

[B87] Sorescu D. Weiss D. Lassègue B. Clempus R. E. Szöcs K. Sorescu G. P. (2002). Superoxide production and expression of nox family proteins in human atherosclerosis. Circulation 105 (12), 1429–1435. 10.1161/01.cir.0000012917.74432.66 11914250

[B88] Steinberg D. (2002). Atherogenesis in perspective: hypercholesterolemia and inflammation as partners in crime. Nat. Med. 8 (11), 1211–1217. 10.1038/nm1102-1211 12411947

[B89] Sun S. Lulla A. Sioda M. Winglee K. Wu M. C. Jacobs D. R. (2019). Gut microbiota composition and blood pressure. Hypertension 73 (5), 998–1006. 10.1161/HYPERTENSIONAHA.118.12109 30905192 PMC6458072

[B90] Tan X. Feng L. Huang X. Yang Y. Yang C. Gao Y. (2017). Histone deacetylase inhibitors promote eNOS expression in vascular smooth muscle cells and suppress hypoxia-induced cell growth. J. Cell Mol. Med. 21 (9), 2022–2035. 10.1111/jcmm.13122 28266122 PMC5571528

[B91] Tang W. H. Hazen S. L. (2014). The contributory role of gut microbiota in cardiovascular disease. J. Clin. Invest. 124 (10), 4204–4211. 10.1172/JCI72331 25271725 PMC4215189

[B92] Tanoue T. Atarashi K. Honda K. (2016). Development and maintenance of intestinal regulatory T cells. Nat. Rev. Immunol. 16 (5), 295–309. 10.1038/nri.2016.36 27087661

[B93] Tayyeb J. Z. Popeijus H. E. Mensink R. P. Konings M. C. J. M. Mokhtar F. B. A. Plat J. (2020). Short-chain fatty acids (except hexanoic acid) lower NF-kB transactivation, which rescues inflammation-induced decreased apolipoprotein A-I transcription in HepG2 cells. Int. J. Mol. Sci. 21 (14), 5088. 10.3390/ijms21145088 32708494 PMC7404194

[B94] Tayyeb J. Z. Popeijus H. E. Mensink R. P. Konings M. C. J. M. Mulders K. H. R. Plat J. (2019). The effects of short-chain fatty acids on the transcription and secretion of apolipoprotein A-I in human hepatocytes *in vitro* . J. Cell Biochem. 120 (10), 17219–17227. 10.1002/jcb.28982 31106471 PMC6767783

[B95] Tayyeb J. Z. Popeijus H. E. Mensink R. P. Plat J. (2021). Butyric acid added apically to intestinal caco-2 cells elevates hepatic ApoA-I transcription and rescues lower ApoA-I expression in inflamed HepG2 cells Co-cultured in the basolateral compartment. Biomolecules 11 (1), 71. 10.3390/biom11010071 33430253 PMC7825706

[B96] Thymiakou E. Zannis V. I. Kardassis D. (2007). Physical and functional interactions between liver X receptor/retinoid X receptor and Sp1 modulate the transcriptional induction of the human ATP binding cassette transporter A1 gene by oxysterols and retinoids. Biochemistry 46 (41), 11473–11483. 10.1021/bi700994m 17887732

[B97] Tian K. Xu Y. Sahebkar A. Xu S. (2020). CD36 in atherosclerosis: pathophysiological mechanisms and therapeutic implications. Curr. Atheroscler. Rep. 22 (10), 59. 10.1007/s11883-020-00870-8 32772254

[B98] Tian Q. Leung F. P. Chen F. M. Tian X. Y. Chen Z. Tse G. (2021). Butyrate protects endothelial function through PPARδ/miR-181b signaling. Pharmacol. Res. 169, 105681. 10.1016/j.phrs.2021.105681 34019979

[B99] Tolhurst G. Heffron H. Lam Y. S. Parker H. E. Habib A. M. Diakogiannaki E. (2012). Short-chain fatty acids stimulate glucagon-like peptide-1 secretion via the G-protein-coupled receptor FFAR2. Diabetes 61 (2), 364–371. 10.2337/db11-1019 22190648 PMC3266401

[B100] Tumurkhuu G. Dagvadorj J. Porritt R. A. Crother T. R. Shimada K. Tarling E. J. (2018). Chlamydia pneumoniae hijacks a host autoregulatory IL-1β loop to drive foam cell formation and accelerate atherosclerosis. Cell Metab. 28 (3), 432–448. 10.1016/j.cmet.2018.05.027 29937375 PMC6125162

[B101] Verhaar B. J. H. Collard D. Prodan A. Levels J. H. M. Zwinderman A. H. Bäckhed F. (2020). Associations between gut microbiota, faecal short-chain fatty acids, and blood pressure across ethnic groups: the HELIUS study. Eur. Heart J. 41 (44), 4259–4267. 10.1093/eurheartj/ehaa704 32869053 PMC7724641

[B102] Wahlström A. Sayin S. I. Marschall H. U. Bäckhed F. (2016). Intestinal crosstalk between bile acids and microbiota and its impact on host metabolism. Cell Metab. 24 (1), 41–50. 10.1016/j.cmet.2016.05.005 27320064

[B103] Wang Y. Xu Y. Yang M. Zhang M. Xiao M. Li X. (2020). Butyrate mitigates TNF-α-induced attachment of monocytes to endothelial cells. J. Bioenerg. Biomembr. 52 (4), 247–256. 10.1007/s10863-020-09841-9 32588186

[B104] Wang Z. Klipfell E. Bennett B. J. Koeth R. Levison B. S. Dugar B. (2011). Gut flora metabolism of phosphatidylcholine promotes cardiovascular disease. Nature 472 (7341), 57–63. 10.1038/nature09922 21475195 PMC3086762

[B105] Wu J. Jiang Z. Zhang H. Liang W. Huang W. Zhang H. (2018). Sodium butyrate attenuates diabetes-induced aortic endothelial dysfunction via P300-mediated transcriptional activation of Nrf2. Free Radic. Biol. Med. 124, 454–465. 10.1016/j.freeradbiomed.2018.06.034 29964168

[B106] Xiong Y. Miyamoto N. Shibata K. Valasek M. A. Motoike T. Kedzierski R. M. (2004). Short-chain fatty acids stimulate leptin production in adipocytes through the G protein-coupled receptor GPR41. Proc. Natl. Acad. Sci. U. S. A. 101 (4), 1045–1050. 10.1073/pnas.2637002100 14722361 PMC327148

[B107] Yamamoto Y. Gaynor R. B. (2001). Role of the NF-kappaB pathway in the pathogenesis of human disease states. Curr. Mol. Med. 1 (3), 287–296. 10.2174/1566524013363816 11899077

[B108] Yan Q. Gu Y. Li X. Yang W. Jia L. Chen C. (2017). Alterations of the gut microbiome in hypertension. Front. Cell Infect. Microbiol. 7, 381. 10.3389/fcimb.2017.00381 28884091 PMC5573791

[B109] Yang H. Mohamed A. S. Zhou S. H. (2012). Oxidized low density lipoprotein, stem cells, and atherosclerosis. Lipids Health Dis. 11, 85. 10.1186/1476-511X-11-85 22747902 PMC3475066

[B110] Yissachar N. Zhou Y. Ung L. Lai N. Y. Mohan J. F. Ehrlicher A. (2017). An intestinal organ culture system uncovers a role for the nervous system in microbe-immune crosstalk. Cell 168 (6), 1135–1148. 10.1016/j.cell.2017.02.009 28262351 PMC5396461

[B111] Zapolska-Downar D. Siennicka A. Kaczmarczyk M. Kołodziej B. Naruszewicz M. (2004). Butyrate inhibits cytokine-induced VCAM-1 and ICAM-1 expression in cultured endothelial cells: the role of NF-kappaB and PPARalpha. J. Nutr. Biochem. 15 (4), 220–228. 10.1016/j.jnutbio.2003.11.008 15068815

[B112] Zeng X. Gao X. Peng Y. Wu Q. Zhu J. Tan C. (2019). Higher risk of stroke is correlated with increased opportunistic pathogen load and reduced levels of butyrate-producing bacteria in the gut. Front. Cell Infect. Microbiol. 9, 4. 10.3389/fcimb.2019.00004 30778376 PMC6369648

[B113] Zhang Q. Fan X. Y. Cao Y. J. Zheng T. T. Cheng W. J. Chen L. J. (2021). The beneficial effects of Lactobacillus brevis FZU0713-fermented Laminaria japonica on lipid metabolism and intestinal microbiota in hyperlipidemic rats fed with a high-fat diet. Food Funct. 12 (16), 7145–7160. 10.1039/d1fo00218j 34231612

[B114] Zhao L. Zhang F. Ding X. Wu G. Lam Y. Y. Wang X. (2018). Gut bacteria selectively promoted by dietary fibers alleviate type 2 diabetes. Science 359 (6380), 1151–1156. 10.1126/science.aao5774 29590046

[B115] Zhao Y. Liu J. Hao W. Zhu H. Liang N. He Z. (2017). Structure-specific effects of short-chain fatty acids on plasma cholesterol concentration in male Syrian hamsters. J. Agric. Food Chem. 65 (50), 10984–10992. 10.1021/acs.jafc.7b04666 29190422

